# αII-spectrin in T cells is involved in the regulation of cell-cell contact leading to immunological synapse formation?

**DOI:** 10.1371/journal.pone.0189545

**Published:** 2017-12-15

**Authors:** Justyna M. Meissner, Aleksander F. Sikorski, Tomasz Nawara, Jakub Grzesiak, Krzysztof Marycz, Dżamila M. Bogusławska, Izabela Michalczyk, Marie-Christine Lecomte, Beata Machnicka

**Affiliations:** 1 Laboratory of Cytobiochemistry, Biotechnology Faculty, University of Wrocław, Wrocław, Poland; 2 Electron Microscopy Laboratory, Faculty of Biology, University of Environmental and Life Sciences Wrocław, Wrocław, Poland; 3 Faculty of Biological Sciences, University of Zielona Góra, Zielona Góra, Poland; 4 Biologie Intégrée du Globule Rouge UMR_S1134, Inserm, Univ. Paris Diderot, Sorbonne Paris Cité, Univ. de la Réunion, Univ. des Antilles, Paris, France; Emory University School of Medicine, UNITED STATES

## Abstract

T-lymphocyte activation after antigen presentation to the T-Cell Receptor (TCR) is a critical step in the development of proper immune responses to infection and inflammation. This dynamic process involves reorganization of the actin cytoskeleton and signaling molecules at the cell membrane, leading to the formation of the Immunological Synapse (IS). The mechanisms regulating the formation of the IS are not completely understood. Nonerythroid spectrin is a membrane skeletal protein involved in the regulation of many cellular processes, including cell adhesion, signaling and actin cytoskeleton remodeling. However, the role of spectrin in IS formation has not been explored. We used molecular, imaging and cellular approaches to show that nonerythroid αII-spectrin redistributes to the IS during T-cell activation. The redistribution of spectrin coincides with the relocation of CD45 and LFA-1, two components essential for IS formation and stability. We assessed the role of spectrin by shRNA-mediated depletion from Jurkat T cells and show that spectrin-depleted cells exhibit decreased adhesion and are defective in forming lamellipodia and filopodia. Importantly, IS formation is impaired in spectrin-depleted cells. Thus, spectrin may be engaged in regulation of distinct events necessary for the establishment and maturity of the IS: besides the involvement of spectrin in the control of CD45 and LFA-1 surface display, spectrin acts in the establishment of cell-cell contact and adhesion processes during the formation of the IS.

## Introduction

**Fig 1:**
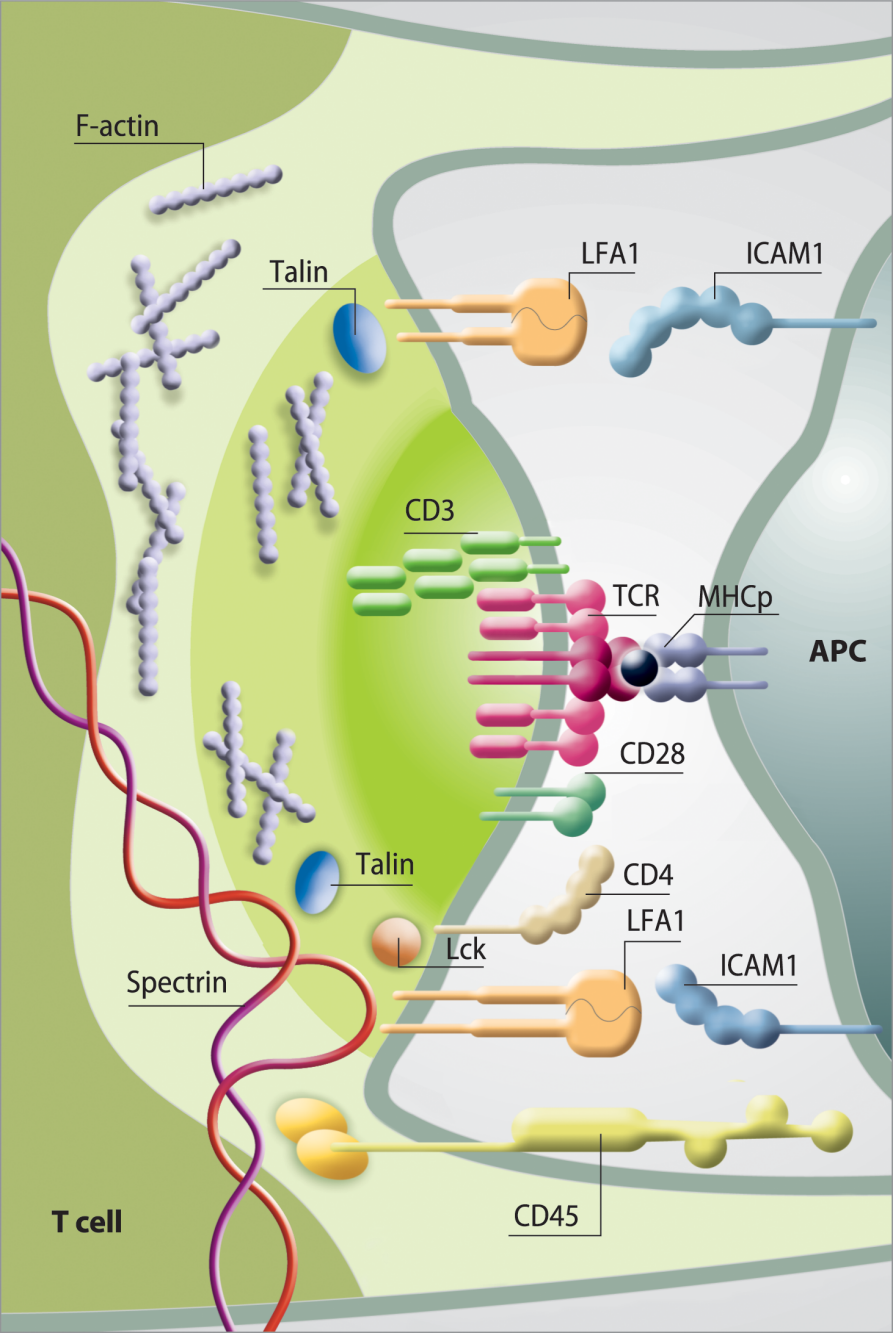
Schematic of the immunological synapse (IS) and representative protein interactions in the synaptic space. Distribution of receptors and adhesion molecules in individual clusters in the immune synapse. The T-cell receptor (TCR) / CD3 complex interacts with MHC-peptide. The adhesion molecules on the surface of both cells (LFA-1—ICAM- 1 are responsible for the formation and stabilization of the IS, as well as for initiating signal transduction pathways activated by TCR. The distal ring of IS is rich in proteins, such as CD45 and F-actin controls lamellipodia and filopodia formation.

Primary lymphocytes are activated *via* a multi-step mechanism that begins with weak adhesion and stimulation of the T-cell receptor (TCR) by antigens exposed on the surface of antigen-presenting cells (APCs). This direct interaction induces a dynamic process that leads to the formation of specialized membrane junctions and adhesion strengthening. The contact site between cells provides a highly organized immunological synapse, a multi-protein signaling apparatus for controlling gene expression [1–3]. All signaling events must be coordinated in time and space to achieve accurate T-cell activation, and each of these activities is dependent on the actin cytoskeleton. Actin drives the process of cell polarization, maintains cell–cell contact and provides a scaffold for clustering, translocation and spatial segregation of proteins, key steps to amplify and sustain T-cell signaling [4]. TCR interactions with CD8 protein on APCs results in increased concentration of the membrane-associated tyrosine phosphatase, CD45, in the central part of the cell-cell contact area [5]. Afterwards, CD45 down regulates the activity of proximal lymphocyte-specific tyrosine protein kinase (Lck), thus modulating early antigen-independent signals leading to actin cytoskeleton rearrangements [6, 7]. Proteins that influence synapse structure, such as F-actin and CD45, are present in the cell-cell contact area not only during the early stage of IS formation but also during the multidimensional construction of a mature synapse [8]. The polarization of actin towards cell contact area is accompanied by recruitment of talin which activates lymphocyte function-associated antigen 1 (LFA-1) [9–11]. LFA-1 participates in immune responses by forming a membrane-junction with Intercellular Adhesion Molecule (ICAM-1/2) when T-cells interact with APCs [12–15] (Fig 1).

Identification of new molecular interactions underlying regulation of the immune response may lead to finding novel strategies targeting the IS for therapy. Treatments targeting the synapse have helped to establish immunotherapy as a mainstream element in cancer treatment. It has been demonstrated that impaired actin polymerization results in CD4+ and CD8+ T cells from patients with chronic lymphocytic leukemia (CLL) exhibiting defective immunological synapse formation with APCs. T cell dependent immune responses protect the host from cancer, but also participate in destructive autoimmunity [16–18].

The spectrin-based network plays a critical role in actin cytoskeleton organization and remodeling. Spectrins are multifunctional proteins involved in the regulation of cell morphology and mechanical properties [19, 20]. Numerous spectrin isoforms are expressed in nonerythroid cells, where the composition of the spectrin network is highly complex and performs different functions [21]. Many data suggest that spectrins directly or indirectly may control the distribution and activity of several proteins engaged in cell-cell contact and signaling, including actin, CD45 and LFA1 [22–25]. It has been proposed that spectrin, as a protein involved in actin skeleton organization, may play an important role in T-cell activation and IS formation.

In this study, we focus on the role of spectrin in T-cell activation, using confocal microscopy analysis combined with advanced electron microscopy techniques. We describe here the effect of spectrin depletion in T-cells early stages of immunological synapse formation i.e. on the dynamics of actin during lamellipodia and filopodia formation and distribution of LFA-1 and CD45 during cell-cell contact organization. Obtained data may suggest a regulatory function of nonerythroid spectrin in the formation of the contact area between the T-cell and an APC.

## Materials and methods

### Antibodies

Rabbit anti- αII and anti- βII spectrin primary antibodies were readily available from the Laboratory of Cytobiochemistry, University of Wroclaw, Poland [eg.[26]].

Rabbit anti-human αII-spectrin (H-105), rabbit anti-human β-actin (I-19), goat anti-human CD45 (N-19), goat anti-human LFA-1 (N-18) were purchased from Santa Cruz Biotechnology, USA. Anti-goat and anti-rabbit Cy2-, Cy5- (Jackson Immunoresearch Laboratories, INC) or Alexa Fluor 568–conjugated (A-11057, Thermo Fisher Scientific) IgG and anti-rabbit IgG conjugated with HRP (Abcam) were used. F-actin was labeled with Alexa Fluor 568-phalloidin (A12380, Thermo Fisher Scientific). Immunogold labeling was performed using 10 nm gold-conjugated anti-rabbit secondary antibodies produced in goat (Electron Microscopy Sciences, USA).

### Cell cultures

The Jurkat T-cell line (German Collection of Microorganisms and Cell Cultures, ATCC^®^ Number TIB-152), HuT 78 (gift from A. Miazek—Institute of Immunology and Experimental Therapy, Polish Academy of Sciences) and primary peripheral blood mononuclear cells (PBMCs) were cultured at 37°C, in 5% CO_2_ and in a humidified atmosphere in RPMI 1640 medium supplemented with 10% FBS, 2 mM glutamine and 100 μg/ml penicillin and streptomycin (Sigma Aldrich, USA).

### White blood cell isolation from peripheral blood

Peripheral blood from healthy volunteers was diluted three times with PBS. The Ethics Committee of the Medical University of Wroclaw approved the study protocol (no. KB-541/2011). Informed consent was obtained from all of the subjects (only adults were involved). White blood cells were obtained by density-gradient centrifugation at 800 g on Ficoll-Paque (density = 1.078; GE Healthcare) for 40 min at room temperature. After centrifugation, the fraction of white blood cells was harvested, counted, and cultured at 37°C, in 5% CO_2_ and in a humidified atmosphere in RPMI 1640 medium supplemented with 10% fetal bovine serum, 2 mM glutamine and 100 μg/ml penicillin and streptomycin (Sigma Aldrich, USA).

### Spectrin knockdown

Two strategies of silencing of the *SPTN1* gene expression were used:

1. Jurkat cells were transfected with short hairpin RNA plasmids (pGeneClip™ hMGFP, SureSilencing™ shRNA, SA Biosciences) expressing GFP and a non-targeting sequence used as control (non-relevant shRNA, named Nr-shRNA) or a sequence targeting the αII-spectrin gene, (αII-spectrin shRNA named Sp-shRNA) using the Cell Line Nuclofector Kits (Amaxa Biosystem) AMAXA, according to the manufacturer’s instructions. The transfection efficiency and cell viability were estimated by flow cytometry (FACSCalibur flow cytometer, BD Biosciences) at 24 h after transfection performed with either negative control shRNA labelled with pmax-GFP and after cell treatment with 5 μg/ml propidium iodide (PI). Three Sp-shRNA plasmids were tested 1, 2 and 3. Depletion efficiency of αII-spectrin expression was estimated by western blot 48 h after transfection.
  Other plasmid expressing the LifeAct peptide fused with fluoro-Ruby (Ruby-LifeAct, a red marker visualizing F-actin in living cells) were also used to transfection Jurkat cells.
2. Cells were transfected with lentiviral particles containing short hairpin RNA plasmids (shRNA, SA Biosciences) expressing a scrambled sequence, used as control (scrambled shRNA, named SC-shRNA), or a sequence targeting the αII-spectrin gene (αII-spectrin shRNA named Sp-shRNA), according to the manufacturer’s instructions (Santa Cruz Biotechnology, Inc. αII-spectrin shRNA (m) Lentiviral Particles: sc-36550-V). Depletion efficiency of αII-spectrin expression was estimated by western blot. The assessment of silencing effect for both strategies are presented in supplementary material (S1 Fig).

### T-cell activation

Activation of T-cells was performed according to two methods. In one approach, polystyrene magnetic beads coated with an optimized mixture of monoclonal antibodies directed against ε chain of CD3 complex and anti-CD28 expressed on the T-cell surface (Dynabeads R Human T-Activator CD3/CD28, Thermo Fisher Scientific) were used to simulate antigen presentation and to activate the T-cells. Magnetic beads washed in PBS containing 5% human serum were mixed with cells at a ratio of 1:1 and incubated in PBS buffer containing 5% human serum at 4°C for 30 min, then for 4 min at 37°C. Cells were cyto-spun onto coverslips (ø12 mm) by centrifugation for 10 min at 800 g.

In the second method, coverslips (ø12 mm) were incubated with 0.01% poly-L-lysine for 24 h at room temperature (RT). At the indicated times, coverslips were washed and coated with anti-CD3 and anti-CD28 antibodies (Miltenyi Biotec) at a dilution of 1:500 by incubation for 1h at room temperature. After washing with PBS buffer, plates were used for seeding Jurkat T-cells (1.2x10^5^).

### Immunofluorescence and confocal microscopy

Cells were fixed for 10 min in 4% paraformaldehyde (PFA), washed and blocked for 1 h in 1% FBS in PBS at room temperature. After this time, cells were permeabilized with 1% Triton X-100 and stained overnight at 4°C with anti-spectrin antibody, anti–LFA-1 antibody and/or anti–CD45 antibody. For visualization in the confocal microscope, the cells were exposed to the secondary antibodies mentioned above. The fluorescence staining of F-actin filaments in cell cultures with Alexa Fluor 568-phalloidin was performed for 40 min at RT. Samples were mounted and counterstained for nuclei with Roti-Mount FluorCare DAPI (1.5μg/ml, Carl Roth GmbH). Observations were performed using a Zeiss Laser Scanning Microscope 510 (LSM510; Carl Zeiss, Jena Germany). ZEN software and apochromat ×60 oil immersion objective lens (numerical aperture 1.4) were used for image acquisition.

### Live-cell analysis

Jurkat T-cells were co-transfected with Ruby-Life Act and either SC-shRNA or Sp-shRNA. Observation of global cell dynamics, contact with Dynabeads coated with anti-CD3 and CD28 antibodies and lamellipodia formation were started at 48 h after transfection. Cells mixed with Dynabeads (1:1 number ratio) were plated in IQ4 slides (Biovalley) at a density of 1x 10^5^ cells/ml and incubated at 37°C in 5% CO_2_. The different events (number and stability of lamellipodia) were followed in the Biostation system (Biostation IM, Nikon), every 5 min for 12 h. Ten fields were analyzed for each experimental condition.

### Static cell adhesion assays

Adhesion assays were performed on culture dishes presenting an activated surface. Control and transfected cells growth in rich medium were plated in triplicate on 12-well plates (10^6^ cells per well) and incubated for 30 min at 37°C in 5% CO_2_. After gentle washing in PBS buffer, adherent cells were counted using Image-ProPlus software. The results are expressed as the mean percentages of spectrin-depleted adherent cells versus control adherent cells.

### Scanning electron microscopy

Cells were fixed by either 4% PFA or 2.5% glutaraldehyde in PBS during 30 min at 4°C. After fixation, the samples were washed three times for 10 min in distilled water. Cell dehydration was performed in 50% ethanol at RT for 10 min, then with increasing ethanol concentrations (60%, 70%, 80%, 90%, 98%). Samples were finally treated with 100% ethanol and then allowed to air-dry overnight. The coverslips were coated with gold using ScanCoat6 (Edwards) and observed via SEM using a SE1 detector, at 10 kV filament tension (SEM, Zeiss Evo LS 15).

### Immunogold labelling and transmission electron microscopy

Cells were fixed in 3.2% paraformaldehyde and 0.5% glutaraldehyde for 10 min at RT and washed twice in Sorensen's buffer (pH = 7.3). Samples were then dehydrated in a graded series of ethanol, each one for 10 min, up to 70% of ethanol. Immunogold labeling was performed using LR-White resin (London Resin Company Ltd) mixed at 2:1 with 70% ethanol for 2x1h, followed by incubation in pure LR-White overnight at RT and in pure LR-White for 48 h at 50°C. Ultrathin sections (~65 nm) obtained using an ultramicrotome were mounted on nickel grids. In order to inactivate aldehyde groups present after aldehyde fixation, grids were incubated in PBSG buffer (50 mM glycine in PBS, pH 7.4) for 20 min, then incubated with the blocking solution (Aurion blocking solution, EMS, USA) for 15 min at RT. After blocking, grids were washed twice with antibody incubation solution (0.2% Aurion blocking solution in PBS, pH = 7.4) and incubated with primary antibodies (rabbit anti-spectrin, 1:20 dilution) overnight at 4°C. After incubation, grids were immunolabeled with 10 nM gold-conjugated secondary antibodies (1:20 dilution) for 1h at RT. After washing in 0.2% Aurion blocking solution in PBS (6×10 min) and distilled water (2×5 min), the grids were post-stained in uranyl acetate and Reynolds reagent for 1 h. The samples were examined using a focused ion beam in an *AURIGA* workstation (FIB-SEM, AURIGA 60, Carl-Zeiss, Germany), 20kV of filament tension bright field.

### Statistical analysis

Each experiment was performed at least in triplicate, and values represent means of at least three independent experiments. Significance was determined using the Student’s t-test, and a 95% confidence interval was set, a priori, as the desired level of statistical significance.

## Results

### Spectrin redistributes to cell-cell contact sites during IS formation

**Fig 2:**
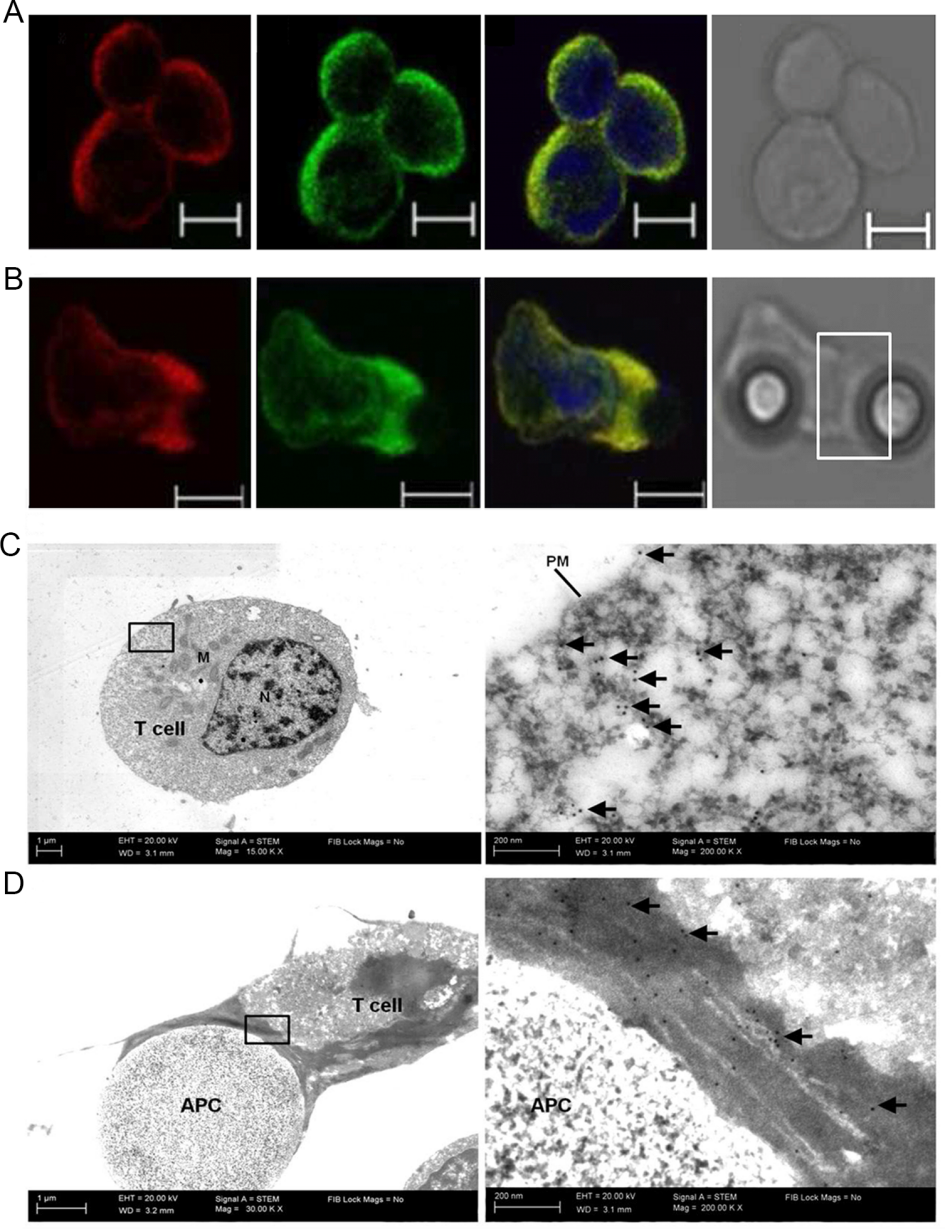
Spectrin translocates together with actin to the IS upon T-cell activation. **(A-B)** Distribution of spectrin (red, 1) and actin (green, 2) in primary T lymphocytes from PBMCs population in the absence (naive) (A) or presence (activated) (B) of Dynabeads coated with anti-CD3 and anti-CD28, along with a merged (3) and a TL(4) image. The white rectangle marks the contact site. The scale bar = 5μm. The results are representative of at least three independent experiments. **(C-D)** Transmission electron micrographs of non-activated (C) and activated with Dynabeads coated with anti-CD3 and anti-CD28 (D) Jurkat T-cells labeled with antibodies directed against spectrin, followed by gold-conjugated secondary antibodies. The right column shows enlarged images of the marked areas. Arrows point to gold particles (spectrin). Almost all of the gold in panel D accumulate at the point of cell-bead contacts. Abbreviations: APC-antigen presenting cell simulated by Dynabeads coated with anti-CD3 and anti-CD28; N-nuclei, PM-plasma membrane, M-mitochondria. Magnification 30 000x, scale bar = 1 μm (200 000x, scale bar = 200 nm), indicated on micrographs.

Stabilization of the contact area between an APC and T-cell requires actin polymerization at the site of the IS [27, 28]. Numerous actin-binding proteins such as PLC-γ1(Phosphoinositide phospholipase C), adaptor NCK (non-catalytic region of tyrosine kinase), ITK (interleukin-2-inducible T cell kinase), Arp2/3 complex (*A*ctin-*R*elated *P*roteins ARP2 and ARP3), WASp (Wiskott–Aldrich Syndrome protein) and Dyn2 (Dynamin-2) have been shown to redistribute to the IS [29–32]. However, the behavior of spectrin has not been explored in this regard. Therefore, we assessed the distribution of spectrin in T-cells during cell-cell contact leading to IS formation. The activation of T-cell by APCs was simulated by using beads coated with antibodies directed against CD3 and CD28. In non-activated primary peripheral blood mononuclear cells (PBMCs), αII-spectrin is mainly observed in a diffuse distribution around the cell perimeter (Fig 2A). Similarly, labeling with phalloidin shows a regular organization of the actin cytoskeleton at the plasma membrane. However, when primary lymphocytes within PBMC population are stimulated for 30 min with APC-simulating Dynabeads coated with anti-CD3 and CD28 antibodies, spectrin and actin are mainly detected at the cell-bead contact sites where they co-localize (Fig 2B). Thus, activation of TCR causes the translocation of spectrin along with actin to the presumed IS.

Identical results of αII-spectrin redistribution as for primary T cells were obtained in Jurkat T-cell line (data not shown), which supports the participation of this protein in IS organization. We used Jurkat T-cells as a model system in subsequent studies not only for convenience but also for several other reasons, the most important being the possibility of obtaining stable cell lines with partially silenced *SPTN1* gene expression.

To more precisely assess the distribution of spectrin in naïve and stimulated Jurkat T-cells, we examined its localization at the ultrastructural level using immunogold labeling and transmission electron microscopy. As shown in Fig 2C, αII-spectrin immunolabelling (arrows point to the gold particles) in non-activated Jurkat T-cells localized mostly at the intracellular side of the plasma membrane. However, when cells were activated with anti-CD3 and CD28, immunolabeling is mainly observed at sites of cell-bead contact (Fig 2D, arrows). These ultrastructural observations are analogous to the level of light-microscopical analyses. Both labelling methods reveal αII-spectrin accumulation at cell-bead contacts in both stimulated T cell populations used, i.e. in primary lymphocytes and Jurkat T cells.

### Knockdown of spectrin in T cells causes a defect in cell adhesion and lamellipodial networks

A link between spectrin and adhesion complex formation has been established previously. Metral and co-workers [22] reported that melanoma cells depleted of αII spectrin exhibit impaired adhesion and modified expression of integrins. In addition, spectrin has an important function in the adhesive properties of neurites and the surface expression of adhesion molecule LFA1 in human neuroblastoma cells [33]. Presented above results revealed the presence of αII spectrin in Jurkat T-cells at sites of IS formation, suggesting possible role of spectrin in this process. One of the key events that occurs during IS formation is increased specific T- cell adhesion. To investigate the putative participation of spectrin in Jurkat T-cell adhesion, spectrin was depleted in these cells using two kinds of shRNA strategy (for details see Materials and Method section). In first, cells were transfected with control shRNA (SC-shRNA) or three different shRNAs targeting human αII-spectrin mRNAs. As shown by western blot analysis, 48 h after transfection, these different shRNAs efficiently silenced αII-spectrin expression by ~60% (S1 Fig). The other strategy was obtaining stable spectrin depleted Jurkat cell line, cells were transduced with lentiviral vectors containing short hairpin RNAs expressing scrambled sequences (SC-shRNA), or αII-spectrin specific sequences (sp-shR,NA) and levels of αIIβII-spectrin were analyzed by Western blotting. As shown in S1 Fig cells transduced with αII-spectrin specific RNA show significantly lower levels of αII-spectrin than control cells, when normalized to cellular actin. Depletion of the αII-spectrin subunit leads to a decrease in the level of βII chain. Morphology and phenotype of these spectrin depleted cells obtained by two different strategies did not show noticeable differences, so even though most of experiments were performed on cells obtained *via* either strategy, most of the results are presented for stable spectrin-depleted Jurkat cell line unless indicated otherwise.

**Fig 3:**
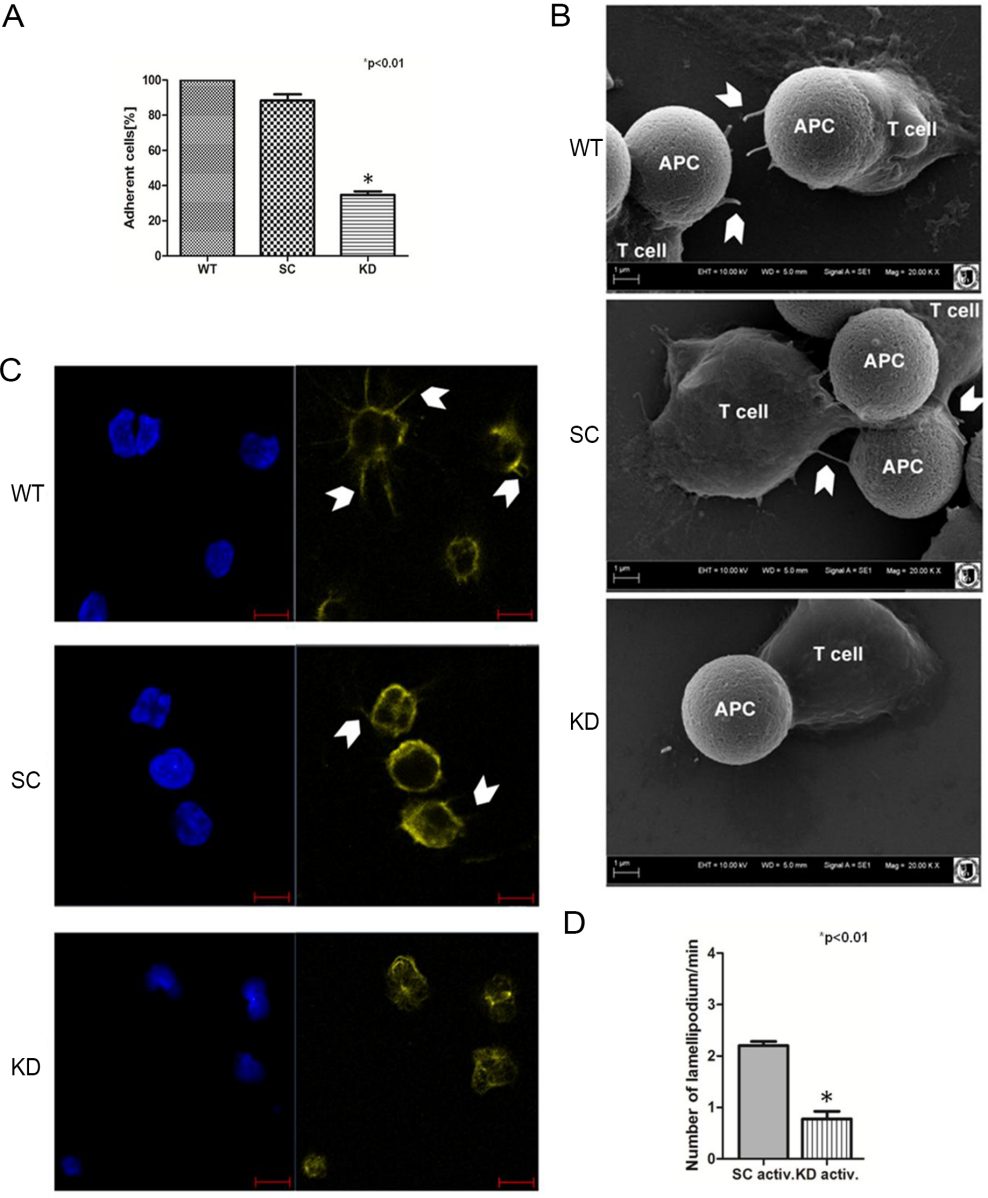
Spectrin depletion impairs lamellipodia formation and cell adhesion. **(A)** Adhesion capability of spectrin-depleted Jurkat T-cells. The data are expressed as the percentage of adherent cells compared with the number of adherent control cells. Data are presented as mean±SD for n = 3, asterisk marks statistically significant (p<0.05), Student’s t test. Abbreviations: WT-Wild type Jurkat T-cells; SC–stable cell line treated with scrambled shRNA; KD–stable cell line treated with anti-spectrin shRNA **(B)** Scanning electron microscopy of contact sites formed by Jurkat T-cells and Dynabeads coated with anti-CD3 and anti-CD28 simulating the APC activation process. Arrowheads indicate lamellipodia formed by T-cells. Magnification 20 000x, scale bar = 1 μm. **(C)** Confocal microscopy of morphological changes and actin (yellow) distribution in T-cells upon IS formation on coverslips coated with anti-CD3 and anti-CD28 antibodies. Nuclei are stained with DAPI (blue). Arrowheads indicate lamellipodia formed by T-cells. Scale bar = 5μm. **(D)** Effect of spectrin knockdown (method A of transfection) on frequency of lamellipodia formation by live-imaging of Jurkat T-cells before and after activation with Dynabeads coated with anti-CD3 and anti-CD28. Cells were co-transfected with Ruby-Life Act plasmid to visualize actin. The frequency of lamellipodia formation and actin dynamics were analyzed in the Biostation system.

To examine the effects of spectrin depletion on adhesion, control cells (wild type, WT), cells transduced with scrambled shRNA (SC) or with anti-spectrin shRNA (KD) were plated on polylysine-coated culture dishes and the number of cells that were adhered to the plate 30 minutes later was quantitated. As shown in Fig 3A only 40% of cells treated with anti-αII-spectrin shRNA adhered, indicating that loss of spectrin significantly decreased the adherence potential of Jurkat T-cells.

The adherence of T-cells to the APCs is facilitated through the formation of lamellipodia. To assess the role of spectrin in the ability of T-cells to form lamellipodia and/or filopodia, we used scanning electron microscopy to analyze the actin lamellipodia at the leading-edge of control and spectrin-depleted T-cells bound to Dynabeads using scanning electron microscopy (SEM). Control Jurkat T-cells (WT) and cells treated with scrambled shRNA (SC) develop numerous lamellipodia and long finger-like projections called filopodia around the Dynabeads coated with anti-CD3 and anti-CD28 (Fig 3B, WT and SC, arrowheads). In contrast, spectrin-depleted cells show much decreased number of lamellipodia and filopodia extensions (Fig 3B, KD).

To confirm and quantitate this effect, lamellipodia formation was assessed by analyzing actin distribution in Jurkat T-cells plated on coverslips coated with antibodies against CD3 and CD28. Control Jurkat T-cells (WT) and cells transfected with scrambled shRNA (SC) were seeded on coverslips coated with CD3 and CD28 antibodies and cultured for 24 hours. Actin staining detected extensive cell spreading and lamellipodia extension, as shown by the thin threads containing actin which protruded from the stimulated cells (Fig 3C, WT and SC, arrowheads). Importantly, depletion of spectrin from cells causes loss of lamellipodia, and induces alterations in cell morphology and shape (Fig 3C, KD). The cells appear much smaller, indicating that spectrin depletion interferes with cell spreading. This is in agreement with the decrease in cell-substrate adhesion of the spectrin-depleted cells, shown in Fig 3A. Similar studies were performed using cell line HuT 78 and comparable effect of spectrin depletion on actin redistribution during activation was observed. Namely, as shown in S2 Fig, activated spectrin-depleted HuT 78 cells did not form lamellipodia and actin did not accumulate in site of cell contact.

Finally, the frequency of lamellipodia formation and their stability were analyzed in Jurkat T-cells by live imaging (S1 and S2 Movies). Cells transfected (according method A) with scrambled shRNA (SC) or with anti- αII-spectrin shRNA were incubated with anti-CD3/CD28-coated Dynabeads and the number of lamellipodia and filopodia formed per 1 minute was quantified during the 12-hour observation period. As shown in Fig 3D, spectrin-depleted cells show more than a threefold decrease in formation of lamellipodia and filopodia compared to control cells.

### Depletion of spectrin in T-cells inhibits the IS formation

T-cell activation depends on the activation of CD45 phosphatase and its accumulation in the cell-cell contact area, an event mediated by its interaction with the spectrin-ankyrin-actin network [1, 6, 7, 23, 34, 35]. Moreover, recent data have shown that adhesion molecules, such as LFA-1, initiate attachment of cells and facilitate the stabilization of the IS [11]. According to recent reports, LFA-1 adhesion enhances actomyosin forces which, in turn, modulate actin assembly downstream of the TCR [36]. Since CD45 and LFA-1 are known to relocate to the IS after T-cell—APC interaction and that we have shown that spectrin also redistributes to the IS, we further examined their possible co-recruitment.

**Fig 4:**
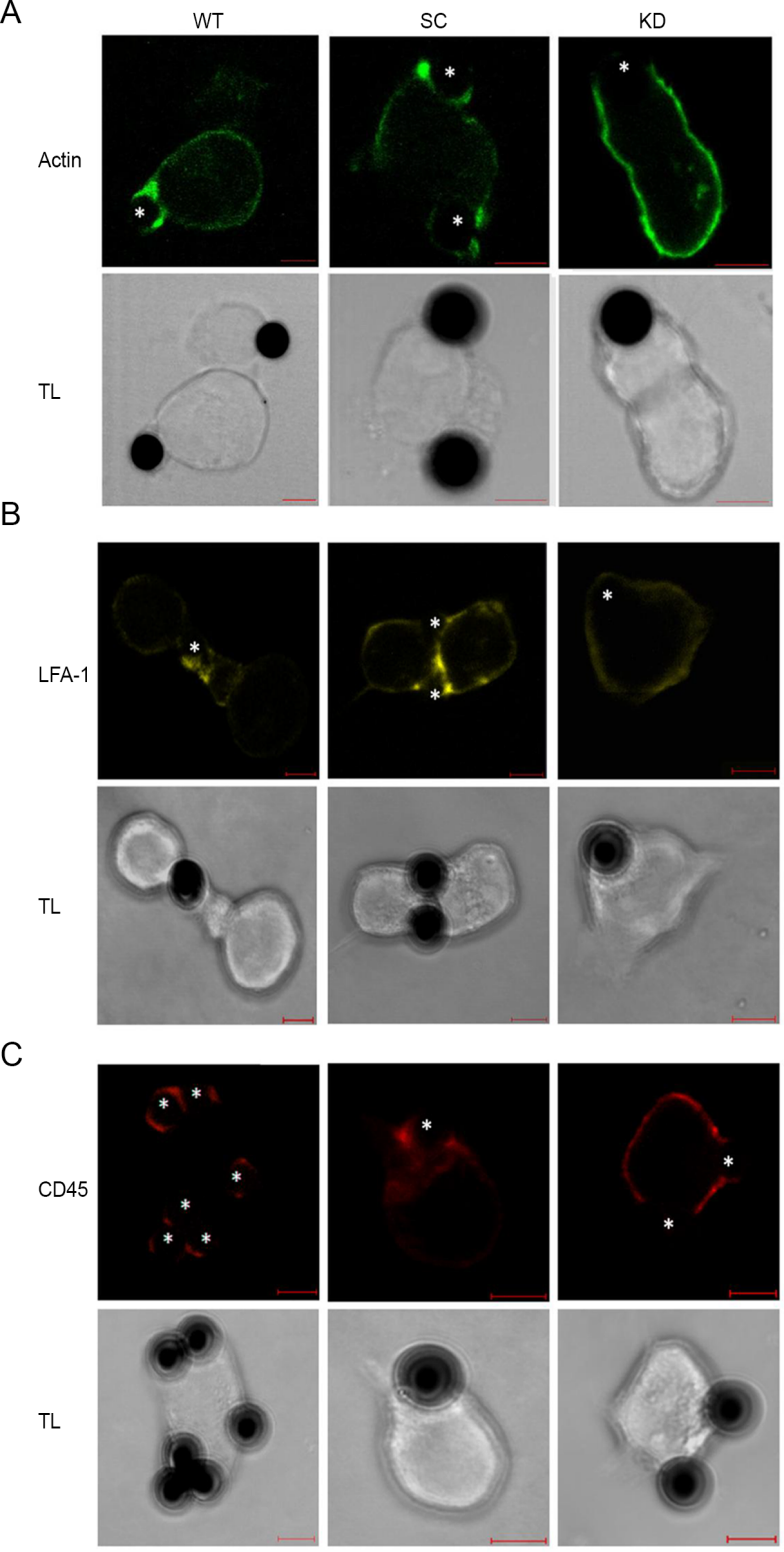
Spectrin depletion leads to impaired IS formation. Confocal images (a single z-plane) of actin (A, green), LFA-1 (B, yellow) and CD45 (C, red) distribution in wild type cells (WT) Jurkat T-cells treated with scrambled shRNA (SC) and cells treated with anti-spectrin shRNA (KD) after activation with Dynabeads coated with anti-CD3 and CD28. Fixed cells were stained with phalloidin-568, anti-LFA-1, or anti-CD45 antibodies. Asterisks indicate the location of the Dynabeads (simulating APC) in the conjugates. The scale bar = 5 μm.

The distribution of actin, CD45 and LFA-1 in non-activated Jurkat T-cells revealed a regular pattern of plasma membrane localization (data not shown). In anti-CD3/CD28 activated cells, accumulation of CD45 and LFA-1 was observed at contact sites between the cells and the beads (Fig 4, WT). Therefore we may conclude, that LFA-1 and CD45 as and αII-spectrin were present in cell-cell contact sites together with actin.

Our results, showing the presence of spectrin at sites of IS in Jurkat T-cells, along with the defects in cell adhesion and lamellipodia formation that we observed in spectrin-depleted cells, raised the possibility that spectrin may participate in IS formation. Thus, we assessed IS formation by following the distribution of actin, LFA-1 and CD45 in control Jurkat T-cells (WT), cells treated with scrambled shRNA (SC) or with anti-αII-spectrin shRNA (KD) after challenging them with Dynabeads coated with anti-CD3 and anti-CD28. As shown in Fig 4, cells transfected with scrambled shRNA (SC) show IS formation at the site of contact with the beads, as evidenced by the redistribution of actin (A), LFA-1 (B) and CD45 (C) into punctate structures tightly concentrated at the contact sites. In contrast, in spectrin-depleted cells, actin remains distributed all over the plasma membrane without any obvious concentration at the site of Dynabead contact (Fig 4A). Similarly, LFA-1 is not recruited to contact sites and remains uniformly distributed all over the plasma membrane in spectrin-depleted cells (Fig 4B). CD45 also shows a defect in redistribution and, although minimal clustering can be seen, the majority of CD45 remains diffusely distributed on the surface of spectrin-depleted cells (Fig 4C). Thus, depletion of spectrin from Jurkat T-cells inhibits the formation of the IS.

## Discussion

Presented here data concerning spectrin involvement in the contact between T-cells and APCs, which is an initial stage of immunological synapse (IS) formation upon T-cell activation, imply a possibility of regulatory role of spectrin in the formation of the cell-cell contact. Cell-cell interactions of this type are a general mechanism to involve some proteins, which leads to the reorganization of the actin skeleton and the formation of specialized cell membrane domains associated with functions that are vital to the body's inflammatory and immune response. Spectrin, a protein of the membrane skeleton family remains in the dynamic equilibrium, both between the cytosol and the membrane and within the membranes (e.g. in the lateral plane of plasmalemma) in connection with the activity of cells, as observed, e.g. during platelet activation or during the formation of intercellular contacts between epithelial cells. Our data may indicate an involvement of spectrin in the process of building intercellular contacts, such as IS.

Actin polymerization in T-cells is critical for several steps of the immune response, including TCR clustering and mature synapse formation [3, 27, 37, 38]. Here, using immune labelling of T-cells, fluorescent and electron microscopy, we observed that upon activation of both the primary T lymphocytes isolated from peripheral blood, in HuT 78 and Jurkat T-cell lines, spectrin and actin rapidly accumulate in the cell-bead contact area, accompanied by their loss from the cytoplasm (see Fig 2 and S2 Fig) what may be a sign of spectrin role in this process.

Published data indicate that spectrin can directly or indirectly interact with proteins involved in cell adhesion [33, 39, 40]. A defect in spectrin in fibroblast cells displayed impaired growth and spreading, a spiky morphology and sparse lamellipodia associated with actin cytoskeleton modifications, such as loss of stress fibres, and modified expression of several integrins [22, 41]. It seems that reduced levels of spectrin in T-cells may also affect cell adhesion as a first stage of IS formation. The results of static adhesion assays of Jurkat cells showed that loss of spectrin reduces the frequency of intercellular contact formation which manifested as significantly reduced number of adherent spectrin-depleted cells compared to control cells (see Fig 3A).

Furthermore, data from confocal microscopy, scanning electron microscopy and live-cell observations support the supposition that the disruption of the spectrin skeleton organisation is associated with a loss of actin-rich lamellipodia in activated spectrin-depleted (KD) Jurkat T-cells (see Fig 3B, 3C and 3D and S1 Movie). Spectrin was found to control the dynamics of actin skeleton indirectly by interaction of the αII subunit SH3 domain with two members of the Ena/VASP family: VASP and EVL [19, 42–45], as well as with Tes, actin binding protein located at cell-cell contact [43]. It also participates in the Rac activation for actin filament formation and spreading [46].

The fact that T-cells isolated from Wiskott-Aldrich syndrome (WAS) patients show characteristic cytoskeletal defects and impaired function, highlights a direct interaction between αII spectrin and testin (Tes), a tumor suppressor [47] localized along stress fibers and participating in cell adhesion [32, 43, 48, 49]. RNA interference knockdown of Tes leads to a loss of actin stress fibers [50]. Moreover, Tes interacts with a variety of cytoskeleton proteins of focal adhesion engaged in IS (such as vinculin and talin)[47, 50].

An equally alternative possibility is that lower level of the αII subunit results in a lower level of the spectrin tetramer, which is the functional form of spectrin and contains an actin-binding domain in its β-subunit (see S1 Fig).

It was shown that αII-spectrin accumulates in specialized integrin clusters that initiate cell adhesion [46, 51]. An intermediate zone of focal adhesion complexes contains force-transduction proteins, such as talin and vinculin, and a stress-fiber interfacial zone that contains actin-associated proteins, such as VASP [52]. We hypothesize that spectrin, through direct interactions with VASP, indirectly controls activation of talin and, in this way, may participate in the regulation of LFA-1 integrin clustering in the IS region.

Binding of LFA-1 on T-cells to ICAM-1 on APCs has been shown to provide a second signal for T-cell activation [51]. This finding may be supported by other data showing that spectrin interacts with many proteins engaged in LFA-1 activation [53]. Furthermore, spectrin by interacting with ankyrin allows CD45 translocation to membrane microdomains during T-cell activation [23, 38]. Data obtained here revealed that, upon stimulation of T-cells, nonerythroid spectrin, CD45 and LFA-1 integrin localizes at the IS region of the plasma membrane indicating that spectrin may be involved in the early steps of IS formation and T-cell activation.

Using shRNA approaches, we have observed that depletion of spectrin in T- cells results in a significant decrease of actin accumulation within the cell-cell contact area. Moreover, in spectrin-depleted T-cells, a pronounced decrease of CD45 and loss of LFA-1 accumulation in the IS area was also observed (Fig 4). The data presented here supports our hypothesis that spectrin may affect the ability of T-cells to form immunological synapses, possibly through interaction with CD45 phosphatase.

In conclusion, our data may indicate a regulatory function of spectrin as a protein involved in first-stages of contact formation between T-cells and APCs. This seems to add another feature to this multifunctional protein of membrane skeleton which was not considered in the past [19, 20, 39]

## Supporting information

S1 Fig **(A)** Jurkat cells were transfected with different shRNA directed against αII-spectrin (Sp-shRNA) or an scrambled shRNA (SC-shRNA). Western Blot analysis revealed a 60% decrease in αII-spectrin expression. Lamin A/C was used to control protein loading. Results revealed efficiency of shRNA1, 2 and 3 on αII-spectrin expression. **(B)** The level of αII- and βII-spectrin in Jurkat T-cells after transduction with lentiviral vectors containing short hairpin RNA plasmids expressing a scrambled control sequence (SC-shRNA) or a αII-spectrin specific sequence (sp-shRNA). A Western Blot was performed with anti- αII-spectrin or anti- βII-spectrin and anti- β-actin (as a loading control). Depletion of αII-spectrin is seen in cells with anti-αII-spectrin shRNA. Depletion of the αII-spectrin subunit leads to a decrease in the level of the βII chain.
(TIF)

S2 Fig Actin redistribution in HuT 78 T cells. Fluorescent microscopy of actin distribution in control (SC) and spectrin-depleted (KD) Hut 78 T-cells in the presence of Dynabeads coated with anti-CD3 and anti-CD28 **(A)** and upon IS formation on plates coated with anti-CD3 and anti-CD28 antibodies **(B)**. Scale bar = 5μm. The results are representative of two independent experiments.
(TIF)

S1 Movie Spectrin depletion impairs cell-cell contact formation. Live-imaging of lamellipodia formation in contact Jurkat T-cells with Dynabeads coated with anti-CD3 and anti-CD28. The spectrin knockdown cells were co-transfected with Ruby-Life Act plasmid to visualize actin.
(AVI)

S2 Movie Live-imaging of lamellipodia formation of control SC Jurkat T-cells. (AVI)
