# Supplementary figures and images for: αII-spectrin in T cells is involved in the regulation of cell-cell contact leading to immunological synapse formation?
Source: PLoS One. 2017 Dec 15;12(12):e0189545. doi: 10.1371/journal.pone.0189545 (PMC5731749; doi:10.1371/journal.pone.0189545)

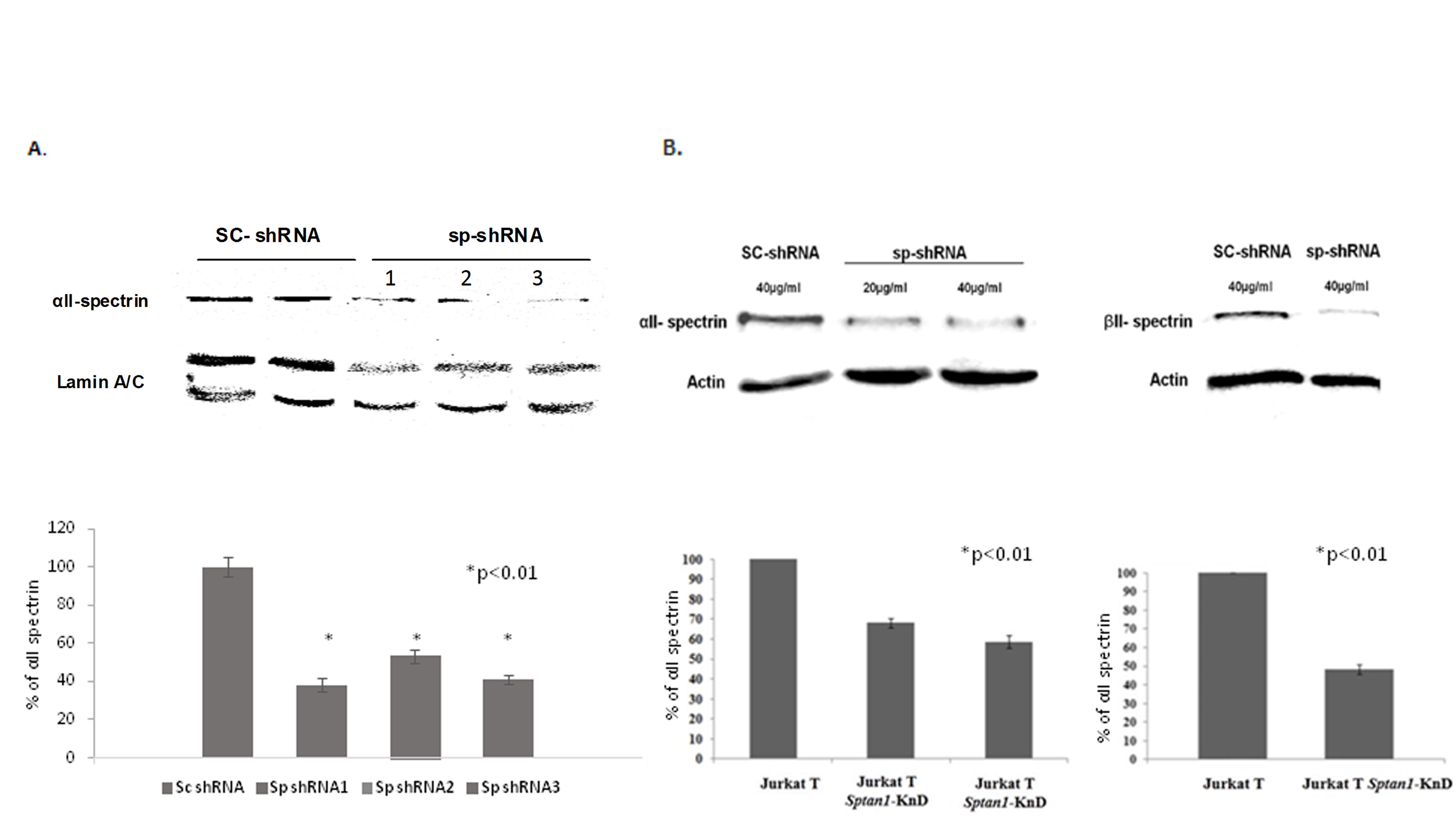

Supplement: S1 Fig — (A) Jurkat cells were transfected with different shRNA directed against αII-spectrin (Sp-shRNA) or an scrambled shRNA (SC-shRNA). Western Blot analysis revealed a 60% decrease in αII-spectrin expression. Lamin A/C was used to control protein loading. Results revealed efficiency of shRNA1, 2 and 3 on αII-spectrin expression. (B) The level of αII- and βII-spectrin in Jurkat T-cells after transduction with lentiviral vectors containing short hairpin RNA plasmids expressing a scrambled control sequence (SC-shRNA) or a αII-spectrin specific sequence (sp-shRNA). A Western Blot was performed with anti- αII-spectrin or anti- βII-spectrin and anti- β-actin (as a loading control). Depletion of αII-spectrin is seen in cells with anti-αII-spectrin shRNA. Depletion of the αII-spectrin subunit leads to a decrease in the level of the βII chain. (TIF) [file pone.0189545.s001.tif]

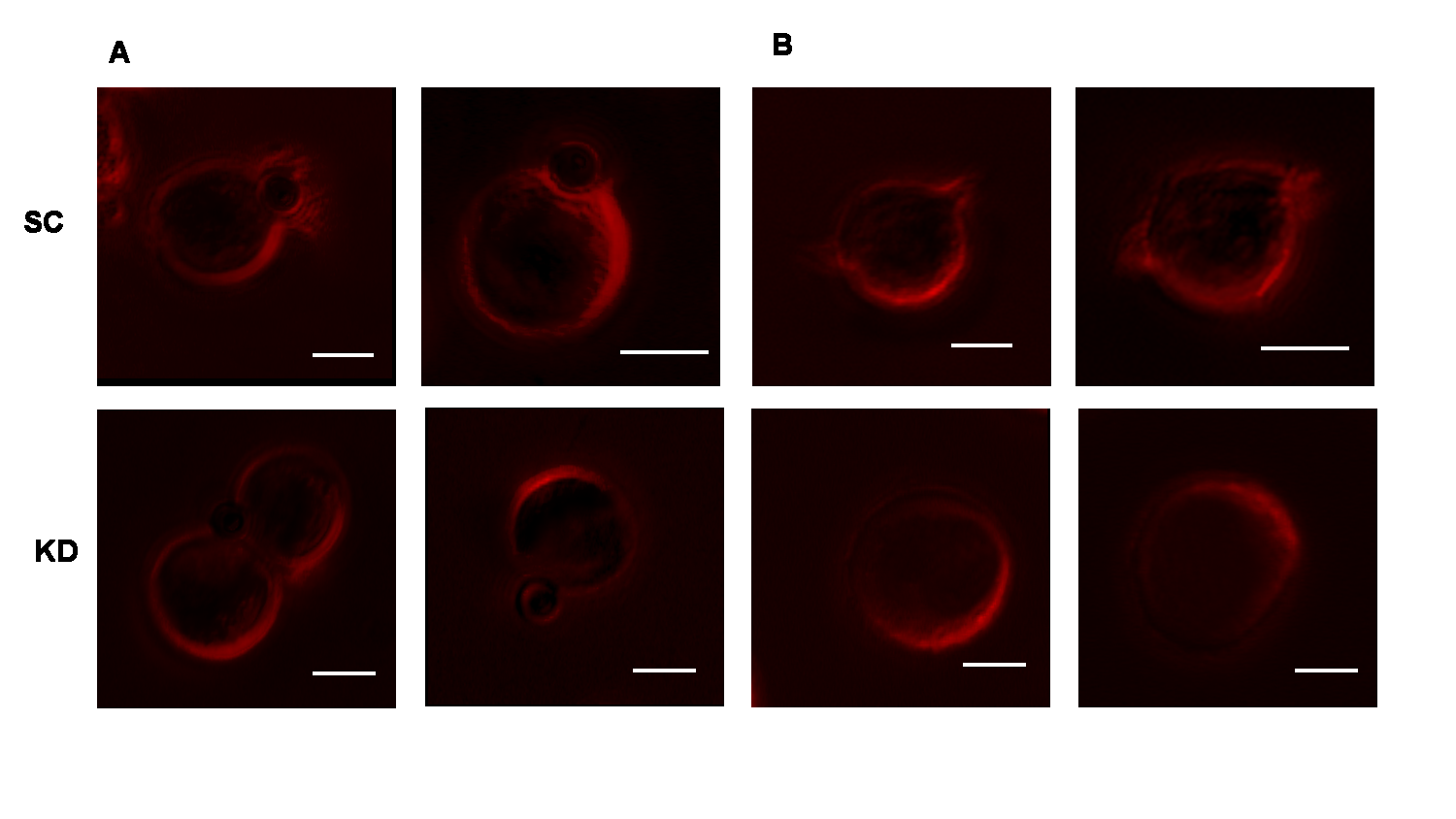

Supplement: S2 Fig — Fluorescent microscopy of actin distribution in control (SC) and spectrin-depleted (KD) Hut 78 T-cells in the presence of Dynabeads coated with anti-CD3 and anti-CD28 (A) and upon IS formation on plates coated with anti-CD3 and anti-CD28 antibodies (B). Scale bar = 5μm. The results are representative of two independent experiments. (TIF) [file pone.0189545.s002.tif]
